# Alloxan-induced diabetes alters rat common carotid artery response to adenosine

**DOI:** 10.1186/2050-6511-13-S1-A16

**Published:** 2012-09-17

**Authors:** Miroslav Radenković, Marko Stojanović, Radmila Janković, Mirko Topalović, Milica Stojiljković

**Affiliations:** 1 Institute of Pharmacology, Clinical Pharmacology and Toxicology, Medical Faculty, University of Belgrade, 11129 Belgrade, Serbia; 2 Institute of Pathology, Medical Faculty, University of Belgrade, 11000 Belgrade, Serbia

## Background

It is well established that diabetes mellitus represents an important risk factor for endothelial dysfunction and associated cardiovascular events. Accordingly, vascular responsiveness of different isolated blood vessels was shown to be altered in experimental diabetes. The aim of this study was to investigate the effect of adenosine on intact or denuded isolated rat common carotid arteries obtained from healthy or diabetic rats.

## Methods

The current study involved two groups of male Wistar rats (220–280 g): (1) healthy controls and (2) rats with alloxan-induced diabetes. Carotid arteries were extracted from rats, carefully dissected from surrounding tissue, cut into 4 mm-long rings and placed in an organ bath. The endothelium was removed from some rings by gently rubbing the intimal surface with stainless steel wire. Apart from the pharmacological verification, the presence of endothelial cells was confirmed by histological evaluation on randomly selected preparations. Concentration-response curves for adenosine (0.01–100 µM) were obtained in a cumulative fashion on serotonin-precontracted arteries.

## Results

The adenosine-induced maximal relaxant response of rings with or without endothelium was similar in all investigated groups (p > 0.05), indicating an equi-effective action of adenosine irrespective of diabetes. The analysis of the median effective concentrations (pEC_50_) showed that the response of intact or denuded vessels to adenosine was comparable but only within each group, thus confirming an endothelium-independent relaxation. On the other hand, the comparison of pEC_50_ values between healthy and diabetic animals showed a significant decrease of pEC_50_ (p < 0.05) in rats with alloxan-induced diabetes, which was also accompanied by a matching rightward shift of the cumulative concentration-response curves for adenosine.

## Conclusions

Adenosine induced endothelium-independent relaxation of the rat common carotid artery, with comparable pharmacological efficacy in all investigated groups, yet with reduced pharmacological potency in diabetic rats. This confirms the initial hypothesis that diabetes alters the response of the rat common carotid artery to adenosine.

